# Zinc Signal in Brain Diseases

**DOI:** 10.3390/ijms18122506

**Published:** 2017-11-23

**Authors:** Stuart D. Portbury, Paul A. Adlard

**Affiliations:** The Florey Institute of Neuroscience and Mental Health, The University of Melbourne, Melbourne, Victoria 3052, Australia; stuart.portbury@florey.edu.au

**Keywords:** zinc, brain, neurodegeneration, cognition

## Abstract

The divalent cation zinc is an integral requirement for optimal cellular processes, whereby it contributes to the function of over 300 enzymes, regulates intracellular signal transduction, and contributes to efficient synaptic transmission in the central nervous system. Given the critical role of zinc in a breadth of cellular processes, its cellular distribution and local tissue level concentrations remain tightly regulated via a series of proteins, primarily including zinc transporter and zinc import proteins. A loss of function of these regulatory pathways, or dietary alterations that result in a change in zinc homeostasis in the brain, can all lead to a myriad of pathological conditions with both acute and chronic effects on function. This review aims to highlight the role of zinc signaling in the central nervous system, where it may precipitate or potentiate diverse issues such as age-related cognitive decline, depression, Alzheimer’s disease or negative outcomes following brain injury.

## 1. Introduction

The essential trace ion zinc is a stable divalent cation that participates in numerous biological processes, and after iron, is the second most abundant trace element [1]. It is a known contributor to the functionality of over 2000 proteins [2], and as such plays a role in many diverse cellular mechanisms including cell division [3], DNA synthesis [4], protein synthesis [5], wound healing [6], immunity [7], and cognition [8]. Thus, zinc is an indispensable micronutrient for humans, the deficiency of which has been linked to numerous disorders [1]. Indeed, zinc deficiency is a recognized global public health concern in developing countries [9], and is also becoming a prevalent concern in the ageing population of developed countries [10]. Whilst supplementation of dietary zinc may be efficacious in the prevention of certain zinc deficiency related conditions, excess zinc can also induce adverse effects due to its role in numerous biochemical reactions in the human body [11]. Thus, the maintenance of zinc at natural homeostatic levels, usually attainable through a balanced diet, is both desirable and essential for optimal physiological function. In the central nervous system (CNS), maintenance of zinc homeostasis is critical for brain health, particularly as it pertains to cognition [12,13]. Moreover, altered zinc homeostasis is considered a contributing factor to the pathogenesis of multiple CNS diseases [14]. In this review, we will provide a brief overview of zinc regulation and will then focus on detailing a number of examples of the role of zinc in different brain diseases.

## 2. Zinc in the Brain

Compared to other organs in the human body, zinc concentration is highest in the brain where it is estimated at 150 μmol/L, representing a 10-fold increase over serum zinc [15]. Zinc occurs in the brain as a structural component of about 70% of proteins, contributing to the efficient performance of over 2000 transcription factors and over 300 enzymes. 10–15% of brain zinc occurs in a “free” or chelatable form, and is present at much lower concentrations (~500 nM) in brain extracellular fluids [15]. However, the concentration of zinc in synaptic vesicles of excitatory glutamatergic forebrain neurons has been shown to be >1 mmol/L [16], and as such has resulted in these cells commonly being referred to as “gluzinergic” neurons. Other cell populations in the brain containing zinc ions in pre-synaptic boutons have similarly been designated as “zinc-enriched” (ZEN) neurons [17].

Zinc homeostasis in the brain is tightly regulated, primarily via three families of proteins. They are the metallothioneins (MTs) involved in the regulation and maintenance of intracellular zinc homeostasis [12], the zinc- and iron-like regulatory proteins (ZIPs) responsible for zinc uptake from extracellular fluids into both neurons and glia [18], and the zinc transporters (ZnTs) which are associated with cellular zinc efflux [1,18]. Whilst historical data has suggested that a number of the ZIP and ZnT proteins were specific for zinc, there is evidence now emerging that suggests that many of these zinc regulatory proteins also regulate other metal ions and are found in a more diverse suite of cellular compartments than originally thought.

## 3. Metallothioneins

Approximately 5–15% of the cytosolic zinc pool is bound by metallothioneins, of which there are four isoforms, designated MT-1, MT-2, MT-3, and MT-4 [19]. Each metallothionein is composed of 61–68 amino acids, comprising 20–21 cysteines which can incorporate up to 7 atoms of zinc for storage, or for the function of a zinc acceptor or donator [1] (these proteins typically also bind copper, and can also bind metals such as cadmium, lead and others). MT-1, MT-2 and MT-3 are all synthesized in the CNS, however MT-1 and MT-2 are expressed in all tissues, and in the CNS are primarily expressed by astrocytes. Similarly, MT-3 is principally expressed in the CNS [19], whereas MT-4 is a minor metallothionein localized in stratified epithelial cells [20].

## 4. Zinc- and Iron-like Regulatory Proteins

There are 14 ZIP transporters currently known in humans [18], each of which contain eight transmembrane domains with extracellular N- and C-termini. Whilst zinc transport activity has been confirmed for ZIPs 1-8 and -14 [21], ZIP transporters do not transport exclusively zinc. They have been shown to regulate intracellular iron, copper, manganese and cadmium transport as well [1]. Most ZIP transporters are localized on plasma membranes where they function in the cytosolic replenishment of zinc from the extracellular space and within the lumen of intracellular components [22]. Cell surface localization and expression of ZIP transporters accordingly increase in zinc-deficient environments, and rapid internalization of the transporters has been observed in conditions of excess zinc [22]. In the CNS, ZIP1 and ZIP3 appear to be the major regulators of zinc uptake, however, ZIP1 expression is greater in the brain than ZIP3, and as such is believed to be the key facilitator of neuronal zinc uptake [22].

## 5. Zinc Transporter Proteins

Currently there are ten known human ZnT proteins, each of which function to regulate zinc primarily via efflux out of cells and intracellular compartments [23]. Whilst there is no precise structural information on ZnTs, based on the structure of the bacterial ZnT homolog YiiP [24], ZnTs are predicted to have six transmembrane domains with cytoplasmic amino- and carboxy-termini.

The first mammalian zinc transporter to be identified and characterized was ZnT1, and it was shown to be primarily localized in the plasma membrane where it functioned by exporting cytosolic zinc ions into the extracellular space [25]. As such, ZnT1 was predicted to protect cells from zinc influx during pathological conditions. Indeed, the mRNA for ZnT1 is upregulated in response to increases in cellular zinc levels in transient forebrain ischemia [26], and to high dietary zinc intake [27]. ZnT2 and ZnT3 both function to transport cytosolic zinc into the lumen of vesicular compartments [23]. ZnT3, which is enriched in the hippocampus and cortex, plays a significant role in modulating neurotransmission and plasticity in glutamatergic neurons [28]. ZnT3 achieves this via its role in loading zinc into pre-synaptic vesicles (whilst other proteins have been reported to perform similar functions, ZnT3 is the primary synaptic vesicular zinc transporter). When zinc ions are then released into the synaptic cleft, which is co-incident with the vesicular release of glutamate [29], it aids in regulating the neuronal processes related to cognition and memory such as long-term potentiation (LTP) and long-term depression (LTD), by acting on *N*-methyl-d-aspartate (NMDA) receptors [30]. Zinc released into the synaptic cleft can also regulate α-amino-3-hydroxy-5-methyl-4-isoxazolepropionic acid receptors on post-synaptic cells, further regulating synaptic function and plasticity [31]. Other interactions with targets such as the zinc sensing receptor (ZnR/GPR39 [32]), the tyrosine kinase receptor TrkB [33], glutamate receptors [34] and p75 [35] also contribute to its role as a modulator of synaptic transmission and plasticity [36] and have led to zinc being labelled as an “atypical neurotransmitter” [37]. These data, together with other detailed studies examining the role of zinc in various aspects of learning and memory in the hippocampus [38,39,40,41,42,43], highlight the significance of zinc in cognition. Significantly, the ablation of ZnT3 has been demonstrated to result in age-dependent cognitive deficits in mice [44]. ZnT3 protein levels are decreased across normal ageing in humans, and further decreased in Alzheimer’s disease postmortem brain tissue [44], together with significant reductions in ZnT3 mRNA levels in disease [45]. Furthermore, there is an emerging notion that single nucleotide polymorphisms in specific genes, such as that for ZnT3 (*SLC30A3*), may have an interaction with the nutrient status that impacts upon short- and long-term memory scores in the normal population [46]. Thus, changes in specific ZnT proteins, or indeed ZIPs or other alterations that alter the normal zinc regulatory apparatus, and that subsequently then precipitate the abnormal homeostasis of zinc within critical brain structures, are likely to alter cognition across both “healthy” and “pathological” ageing. ZnT4 is expressed in the endosomes/lysososmes, Golgi apparatus and trans-Golgi network (TGN) and cytoplasmic vesicles, in the brain, mammary glands and intestinal epithelial cells, where it is involved in vesicular secretory functions [1,23]. ZnT5 and ZnT6 uniquely form heterodimer complexes and function by delivering zinc into early secretory pathways, and both are located in the Golgi apparatus and TGN [23]. ZnT7 is expressed in the large and small intestine, and as such is involved in the absorption of dietary zinc, evidenced through poor growth and reduced adiposity in mice with a disruption in the ZnT7 gene [1,23]. ZnT8 is a pancreatic specific zinc transporter localized to the membrane of insulin storage granules [27]. ZnT9 has been classified as a zinc transporter, however, due to an absence of essential histidine residues, is believed to have no zinc transport functions [1]. ZnT10 has been localized to early/recycling endosomes or the Golgi apparatus, and data suggests that the primary function of ZnT10 is manganese transport. Indeed, patients with homozygous mutations in the ZnT10 gene show disturbances in cellular manganese homeostasis, not zinc perturbations [1].

In the brain, there is a low expression of ZnT2, 5, 7, and 8, however ZnT1, 3, 4, and 6 are highly expressed [23]. Whilst each of these proteins are essential in the modulation of intracellular cytosolic zinc in the brain, it should be noted that ZnT1 and ZnT3 are unique with regards to the other ZnTs in that ZnT1 is present on plasma membranes and ZnT3 is located on synaptic vesicle membranes, with the latter playing a significant role in cognition in aging and pathogenesis of disease.

## 6. Zinc Signal in Brain Diseases

There exists a wide-range of neurological diseases where zinc homeostasis is impacted and subsequently associated with the pathogenesis of the disorder. These include Alzheimer’s disease (AD), amyotrophic lateral sclerosis (ALS), traumatic brain injury (TBI), depression, schizophrenia (SCZ), and Parkinson’s disease (PD). The role of zinc in each of these disorders will be briefly discussed in the following sections.

### 6.1. Alzheimer’s Disease

Alzheimer’s disease is the most common progressive dementia affecting the elderly today. It is a multi-factorial disease of both genetic and non-genetic aetiology. The principal theory of AD pathogenesis has focused on the accumulation of amyloid-β (Aβ), a cleavage product of the much larger amyloid precursor protein (APP). Significant efforts have also focused on the role of abnormally phosphorylated forms of the microtubule associated protein tau in the onset and progression of AD.

The interest of zinc in AD pathogenesis is derived from the observation that zinc, above 300 nM concentration, can precipitate Aβ to result in its aggregation into senile plaques, one of the major pathological hallmarks of the disease [47]. The extracellular concentration of zinc during synaptic transmission rises to 300 μM, and as such it is possible that synaptic transmission could contribute to Aβ deposition in AD [48]. More recently, Deshpande and colleagues [49] also demonstrated that the zinc emanating from the glutamatergic synapse (following neurotransmission) was critical for the targeting of Aβ oligomers to the synapse (where the Aβ subsequently colocalised with the NMDA receptor subunit NR2B). Supporting this notion is the observation that β-amyloid deposits are pronounced in the neocortex, an area in which the highest zinc concentration occurs [48]. The amyloid plaques are also enriched in metals such as zinc [50,51], and Aβ itself is a metalloprotein containing binding sites for metals such as zinc [52]. Further evidence of zinc involvement in Aβ deposition in AD arose from the observation of a significantly reduced plaque load in the brains of Tg2576 transgenic mice (a common mouse model of AD that overexpresses mutant human APP) that were cross-bred with ZnT3 knockout mice, indicating that synaptic zinc does indeed contribute to amyloid deposition in the TG2576 mouse [53]. In contrast to this, plaque number and size have been shown to be enhanced by dietary zinc modulation (both by zinc supplementation, which also resulted in cognitive deficits [54,55], and severe zinc deficiency) in various transgenic mouse models of AD [56,57]. These seemingly paradoxical data may reflect the diverse roles played by zinc in the regulation of Aβ—as zinc has been shown to prevent the proteolytic degradation of Aβ by matrix metalloprotease 2 [58], and also to modulate the activity of α, γ and β-secretase [59] that processes APP to generate Aβ and various cleavage products. Together with the impact of zinc on other pathways, and indeed on other metals (such as copper) that are reported to be involved in the evolution and progression of AD-like neuropathology, it is clear that there is not a simple linear relationship between zinc and Aβ/AD. These observations are not definitive, however, with other studies suggesting a minor role for Aβ and zinc binding [60]. The apparent effect of zinc on modulating the toxicity of Aβ also appears to be concentration dependent, with low concentrations being protective [61]. Similarly, post mortem analysis of AD brain zinc concentrations was contradictory with several studies showing increased zinc levels in the AD brain [50,62], decreased levels in the AD brain [63,64], or no change at all [65]. 

There is an additional pathway through which zinc may be involved in the pathogenesis of AD, which involves its role in the development of the hyperphosphorylated tau protein and causing the polymerization and subsequent generation of neurofibrillary tangles (NFTs), the other major neuropathological feature of the AD brain. Zinc has been revealed to regulate phosphorylation of tau protein through the extracellular signal-regulated kinase pathway (MAP/ERK) [66]. Additionally, low micromolar zinc concentrations can cause the aggregation of human tau fragments [67,68,69], and in rat hippocampal slices synaptically released zinc has been demonstrated to promote the hyperphosphorylation of tau [70]. Recent studies have also demonstrated that zinc inactivates the major tau phosphatase, protein phosphatase 2A (PP2A), via an Src-dependent phosphorylation of PP2A to result in the hyperphosphorylation of tau [71]. Furthermore, zinc may bind to and directly mediate the toxicity of tau [72]. Adding validity to these observations is the fact that the use of zinc chelators or a blockade of synaptic zinc signaling abolishes zinc mediated tau hyperphosphorylation [70]. Cumulatively, these data support an interaction of zinc with the two key proteins, and their regulatory pathways, that are believed to drive disease pathogenesis in AD. This has been the subject of numerous reviews over the last two decades [48,73,74].

### 6.2. Amyotrophic Lateral Sclerosis

Amyotrophic lateral sclerosis (ALS) is a fatal neurodegenerative disease of the human motor system. It is both sporadic and familial in nature with around 5–10% of cases being familial and 90% sporadic. The most common cause of familial ALS is a mutation to the copper, zinc superoxide dismutase (*SOD1*) gene [75]. The mutation, which results in the SOD1 protein having a reduced affinity for zinc, leads to a toxic gain of function in motor neurons when zinc is missing from its active site [76]. In addition to the *SOD1* mutation, both ZnT3 and ZnT6 are downregulated in the spinal cords of patients with sporadic ALS, independent of the loss of motor neurons, suggesting that ZnTs may also have a role in disease pathogenesis [77]. A parallel study in *SOD1* (G93A) mutant transgenic mice, however, indicated that ZnT3 and ZnT6 protein levels were not altered in the mouse spinal cord before or after the onset of ALS symptoms when compared with controls [77]. In another study, it has also been shown that the levels of zinc are significantly higher in the cerebrospinal fluid (CSF) of patients with ALS [78], however, the precise mechanism underlying this elevation and the potential implications for this in disease are yet to be clarified.

### 6.3. Traumatic Brain Injury

Traumatic brain injury (TBI) is a disruption in the normal structure and/or function of the brain that can be attributed to a blow or jolt to the head, or by a penetrating head injury. TBI can destroy neurons via direct mechanical damage such as cellular membrane rupture and diffuse axonal injury (DAI), and indirectly through the ischemia [79]. The ischemia associated with TBI (and ischemic stroke) initiates a release of glutamate from pre-synaptic axon terminals after injury, leading to excitotoxicity and cell death of post-synaptic neurons [80]. Evidence also suggests that the synaptic release of zinc from presynaptic boutons can additionally cause injury and death to post synaptic neurons under excitotoxic conditions. Research findings in both ischemia [81] and status epilepticus [82], both of which occur as secondary complications in TBI [83,84], clearly demonstrate that zinc is translocated from zinc-containing presynaptic boutons to dying postsynaptic soma. In TBI, synaptically released zinc has been shown to contribute to neuronal injury [85], and occurs concomitantly with the glutamate release observed after head injury [86]. However, the role of zinc in the injured brain is not yet clearly defined, with multiple chelation [87,88] and supplementation [89,90] studies demonstrating both neuroprotective and neurotoxic roles for zinc in the pathogenesis of TBI. However, in a rat brain model of TBI, protective effects of zinc chelation were shown to be associated with the upregulation of neuroprotective genes in combination with decreased neuronal death, potentially indicating a toxic role of zinc in TBI [88].

### 6.4. Depression

Depression affects millions of individuals world-wide, and is comorbid with many neurodegenerative diseases [91]. The evidence for a role of zinc in depression has gained much traction over the last decade after the observation that depressed patients exhibited lower serum zinc levels than psychiatrically normal controls, and that zinc levels negatively correlated with the severity of depressive symptoms [92]. Moreover, other studies indicated that serum zinc levels may be normalized after successful antidepressant therapy [93,94]. One explanation as to the role of zinc in depression is its ability to regulate the NMDA receptor [95]. As noted previously, zinc can act as an antagonist to NMDA receptors, and studies of depression have shown that zinc and other antagonists of the NMDA receptor show antidepressant-like effects [96]. Rodent models of depression also support the notion that zinc and NMDA receptors are intimately involved in depression. A recent study by Szewczyk et al., indicated that zinc pre-treatment negated depressive features in the forced swim test, whereby it was observed that zinc treated rodents exhibit longer periods of escape behavior before immobility [97]. Moreover, other studies have demonstrated that zinc administration in rodents can reduce the number of NMDA receptor complexes, indicative of downregulation [98,99]. Other studies have examined the interaction of zinc in relation to the monoaminergic theory of depression, focusing on the serotonergic, noradrenergic and dopaminergic systems [100].

### 6.5. Schizophrenia

Schizophrenia (SCZ) is a long-term mental disorder typified by a breakdown in the association between thought, emotion, and behaviour, leading to faulty perception, inappropriate actions and moods, withdrawal from reality into fantasy, and delusion. It is a condition with both neurodegenerative and neurodevelopmental pathologies with potential causation from maternal zinc deficiency and genetic risk factors [101]. Supporting the notion of the contribution by maternal zinc deficiency is that prenatal zinc deficiency in rodent models produces decreased brain volume [102]. In human SCZ patients, there is a 30–50% reduction in brain zinc content demonstrated for early onset cases compared to control samples in postmortem brain tissue [103,104]. Whilst pre-natal zinc deficiency may not be solely causative of SCZ, interactions with other risk genes, and/or ongoing zinc deficiency following birth may be a contributing factor.

Recently, a genome-wide association study between SCZ and control patients revealed a nonsynonymous single nucleotide polymorphism (nsSNP) in the ZIP8 gene as a risk factor for SCZ [105]. Its direct effect upon zinc transport in SCZ, however, has yet to be elucidated. Additionally, allelic variants in the ZnT3 gene, *SLC30A3*, revealed an increased and gender-specific effect of allele on the risk of SCZ in females [106,107]. Similarly, another study demonstrated an increased cortical expression of the zinc transporter SLC39A12. The expression microarray study revealed messenger RNA (mRNA) for solute carrier family 39 (zinc transporter). Member 12 (*SLC39A12*) was higher in the dorsolateral prefrontal cortex from subjects with SCZ in comparison with controls [108]. These results suggest that a breakdown in zinc cellular homeostasis is likely a part of the pathophysiology of schizophrenia.

### 6.6. Parkinson’s Disease

Parkinson’s disease (PD) is a long-term degenerative disorder of the CNS that mainly affects the motor system. Symptoms develop slowly over time, the most obvious being shaking, rigidity, slowness of movement and difficulty with walking. Both thinking and behavioral problems can occur, and dementia is not uncommon at the advanced disease stage.

There is an observed clinical zinc deficiency in patients presenting with PD [109,110], and while scientific evidence of zinc supplementation in PD patients cohorts are scarce, other animal models of PD demonstrate the efficacy of zinc supplementation. A Drosophila melanogaster PD disease model in which the orthologue of the human Parkin gene was disrupted, was shown to have beneficial responses to zinc supplements [111]. Parkin mutant flies exhibit muscle abnormalities, locomotor defects, an inability to fly owing to the degeneration of indirect flight muscles, as well as a severely reduced lifespan, and as such mimic human PD symptoms. Zinc supplementation, however, ameliorated these deficits. In humans, early onset PD is associated with a mutation of *PARK9*, a lysosomal type 5 P-type ATPase, which has been shown to lead to a reduction of lysosomal zinc storage with an increase in cytosolic zinc and α-synuclein accumulation, a pathological hallmark of the disease [112,113]. Additionally, in PD patients there is an observed accumulation of zinc in the substantia nigra, caudate nucleus and lateral putamen areas associated with PD pathology [114]. Taken together, these data suggest that zinc homeostasis is distorted in PD, and as such may play a contributory role to the pathogenesis of the disease.

## 7. Conclusions

As noted in this review, irregularities in zinc homeostasis may represent a point of intersection for both the pathogenesis and the symptoms that characterize multiple neurodegenerative disorders, in addition to potentially also being involved in the ageing process and associated cognitive decline. If zinc is indeed critical to such a breadth of conditions, then understanding how and why zinc levels change across age and/or prior to or during disease is key to providing the insight necessary to harness the therapeutic potential of zinc (and conversely, to avoid any complications arising from potential zinc toxicity). For example, do zinc levels change because of dietary insufficiency (or perhaps some other aspect of diet or a disease state that alters the absorption of zinc)? Is it due to an age- or disease-related change in zinc importer/transporter levels or function that alters the distribution of zinc? Or perhaps some other change in a different metal or aspect of the metalloproteome that adversely impacts zinc? Is there just one or multiple zinc signaling pathways that are impaired? All these questions and many more require thorough interrogation in order to optimize a zinc-based targeted therapy (e.g., at what stage might zinc supplementation versus chelation be optimal in a given disease state; if it is supplementation, then is bulk dietary modulation sufficient, or is a more targeted pharmacological approach required?). Answers to these questions will ultimately need to be validated in a human clinical trial in order to gain the burden of proof necessary for the wide spread acceptance of zinc as a critical player, and therapeutic target, in disorders of the CNS.
